# Ocular Symptoms Among COVID-19 Positive Patients in Saudi Arabia: A Cross-Sectional Survey

**DOI:** 10.7759/cureus.76671

**Published:** 2024-12-31

**Authors:** Nayef F Alswaina, Abdulrahman Alsowinea, Yazeed K Alhabeeb, Abdulrahman Aljurbua, Asim Alghelfes, Bedr Aljabaan, Hussam S Alshetwi

**Affiliations:** 1 Department of Ophthalmology, College of Medicine, Qassim University, Buraydah, SAU; 2 College of Medicine, Qassim University, Buraydah, SAU

**Keywords:** covid-19, eye manifestation, ocular manifestations, ophthalmology, saudi arabia

## Abstract

Background

Coronavirus disease (COVID-19), a widespread viral illness, has been linked to a range of respiratory and other systemic symptoms. Along with the respiratory symptoms caused by severe acute respiratory syndrome coronavirus (SARS-CoV-2), many extrapulmonary manifestations have also been reported. This study was conducted to report the ocular manifestations of COVID-19 in confirmed cases from the Qassim region, of Saudi Arabia.

Methods

In this retrospective survey-based study, an electronic survey was distributed via social media to individuals who reported a positive COVID-19 test. Demographic data, medical and ocular history, and data about their COVID-19 infection and ocular symptoms were collected.

Results

A total of 200 survey responses were included (35% male and 65% female, age 30.3 years). At least one ocular symptom was reported by 41 (20.5%) participants. Light sensitivity (8.5%), blurred vision (7.5%), redness (7.0%), and eye pain (7.0%) were the most common ocular symptoms.

Conclusion

Among the study participants, ocular symptoms were reported by more than one-fifth of the COVID-19 patients. These ocular symptoms were mostly mild. Further research is required to fully understand the association between COVID-19 and ocular manifestations.

## Introduction

Coronavirus disease (COVID-19) is an infectious disease caused by the severe acute respiratory syndrome coronavirus (SARS-CoV-2). The World Health Organization (WHO) acknowledged COVID-19 as a global pandemic in March 2020. The virus was first noticed in Wuhan, China [1]. Globally, until June 2022, there were 532 million confirmed cases of COVID-19, including six million deaths reported to the WHO and 11 billion vaccine doses administered [2]. The causative agent of COVID-19 is an enveloped beta-coronavirus with a single-stranded positive RNA genome [3]. SARS-CoV-2 binds to the angiotensin-converting enzyme 2 (ACE2) receptor, which is found mainly in the mucosa of the lung, mouth, eyes, gastrointestinal tract, and kidneys, and causes various symptoms such as myalgia, cough, dyspnea, and diarrhea [3,4].

SARS-CoV-2 is transmitted primarily by respiratory droplets when in close contact with infected individuals and indirectly through infected surfaces [5]. Our understanding of other means of transmission is limited, but current evidence suggests that the virus can be transmitted through different means, including what we already mentioned [3,5,6]. While the majority of COVID-19 patients are asymptomatic or experience mild symptoms, some patients develop life-threatening acute respiratory distress syndrome [7]. Prognosis is poor in infected older individuals, men, and patients with comorbidities including hypertension, diabetes, cardiovascular disease, chronic renal disease, hepatic disease, and lung disease [7-9].

SARS-CoV-2 is known to cause respiratory signs/symptoms; however, many extrapulmonary manifestations have been reported, including gastrointestinal tract disturbances and cardiovascular, neurological, and renal manifestations [10,11]. Any tissue expressing ACE2 receptors in their cell wall is prone to being damaged by SARS-CoV-2, and since ACE2 receptors are expressed on the ocular surface, ocular symptoms may be seen in COVID-19 patients. The prevalence of ocular symptoms in confirmed cases ranges from 2% to more than 60% [11,12]. In this study, we investigated the prevalence of ocular manifestations related to COVID-19 reported by individuals in the Qassim region, Saudi Arabia, who tested positive for the disease via polymerase chain reaction. 

## Materials and methods

The sample was collected via an online survey questionnaire developed using the Google Forms platform and distributed to the public via social media (Appendix 1). The survey form was constructed by the authors, and the items of the questionnaire were developed based on a literature review. The survey was open to all individuals who were 18 years of age and older who required to provide their consent to complete the survey. All participants were confirmed to test positive for COVID-19, and they had to be residents of the Qassim region, Saudi Arabia. The study was conducted at Qassim University, Buraydah, Saudi Arabia.

According to the Saudi Ministry of Health, and after the analysis of the data of the COVID-19 positive patients, it was estimated that 350 COVID-19 positive patients' responses are needed. After obtaining ethics approval from the Committee of Research Ethics of Qassim University, survey responses were collected from November 2022 through March 2023. All responses were kept anonymous, and the confidentiality of participant data was maintained. Demographic data, medical and ocular history, and data about their COVID-19 infection and ocular symptoms were collected as study variables. The questionnaire was distributed among large numbers of the population. We gathered around 200 responses, and after the revisions of the results, the response rate was 53%. Data were analyzed using the IBM SPSS Statistics for Windows, Version 21 (Released 2012; IBM Corp., Armonk, New York, United States) and MS-Excel (Microsoft Corporation, Redmond, Washington, United States) statistical packages. Spearman’s correlation analysis was applied to check whether there was a significant association between the study variables. A p-value of ≤ 0.05 was considered to indicate statistical significance.

## Results

Data from 200 participants who met the study inclusion criteria were collected. The study population comprised 130 women (65%) and 70 men (35%). They were between 19 and 67 years old (mean 30.3 years), and more than half (57.0%) of the participants were 19-28 years old (Table 1).

**Table 1:** Demographic characteristics of the study population (N=200).

Characteristic	Frequency (f)	Percentage (%)
Age (years)		
19 - 28	114	57.0
29 - 39	46	23.0
40 - 49	25	12.5
50 - 59	12	6.0
60 - 69	3	1.5
Gender		
Male	70	35.0
Female	130	65.0

**Figure 1:**
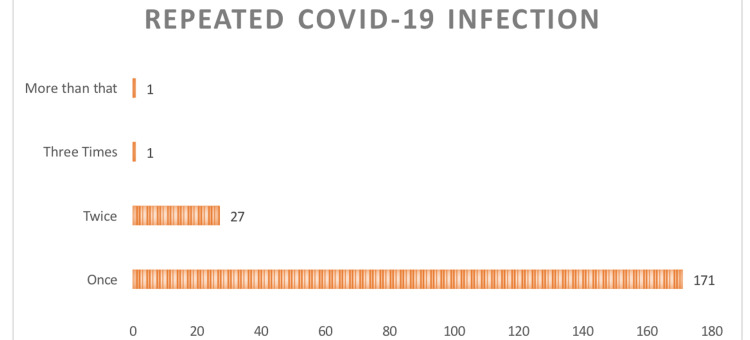
Number of times infected with COVID-19 (N=200)

The participants were asked about the number of times they had been infected with COVID-19. Figure 1 shows that the majority of respondents (85.5%) were infected once, and some were infected twice or more. 

Most participants (n=175) reported a negative history of previous medical or ocular diseases. About 15.0% of participants reported being diagnosed with at least one of the following conditions: diabetes (5.0%), hypertension (4.0%), previous eye surgery (3.0%), or hospital admission because of COVID-19 (3.0%).

At least one ocular symptom was reported by 41 (20.5%) participants. The number of ocular symptoms related to COVID-19 ranged between one and eight symptoms, and 14 (34.1% of participants among those who experienced ocular symptoms) confirmed that they had two symptoms. The 12 (41.4%) participants who were infected once with COVID-19 experienced two types of ocular symptoms, whereas the four (40.0%) participants who were infected twice with COVID-19 experienced one type of ocular symptom (Table 2).

**Table 2:** Distribution of participants with COVID-19 who reported ocular symptoms according to the number of symptoms and frequency of infection (n=41)

No. of COVID-19-related ocular symptoms	Infection frequency									
	Once		Twice		Three times		More than three times		Overall	
	f	%	f	%	f	%	f	%	f	%
1	6	20.7	4	40.0	-	-	-	-	10	24.4
2	12	41.4	2	20.0	-	-	-	-	14	34.1
3	5	17.2	1	10.0	-	-	1	100.0	7	17.1
4	2	6.9	1	10.0	-	-	-	-	3	7.3
5	2	6.9	1	10.0	1	100.0	-	-	4	9.8
6	2	6.9	-	-	-	-	-	-	2	4.9
8	-	-	1	10.0	-	-	-	-	1	2.4
Total	29	100.0	10	100.0	1	100.0	1	100.0	41	100.0

The most common ocular symptoms reported by the participants were light sensitivity (n=17, 8.5% of all responders), blurred vision (n=15, 7.5% of all responders), and eye redness and eye pain (n=14, 7.0% of all responders each). Table 3 shows a summary of ocular symptoms related to COVID-19 infection. Only 12.2% of participants with ocular symptoms (five out of 41 participants) sought medical attention from an ophthalmologist for managing the symptoms.

**Table 3:** Distribution of participants with COVID-19 who reported ocular symptoms according to the number of symptoms and infection frequency (n=41) *More than one response was allowed for this question (total no. of responses was 110) **Overall percentage calculated to the whole sample size (N=200)

Type of COVID- 19-related ocular symptoms	Repeated Infection*									
	Once		Twice		Three times		More than three times		Overall**	
	f	%	f	%	f	%	f	%	f	%
Dryness	8	4.0	3	1.5	-	-	-	-	11	5.5
Redness	7	3.5	7	3.5	-	-	-	-	14	7.0
Burning sensation	8	4.0	2	1.0	-	-	-	-	10	5.0
Double vision	1	0.5	-	-	-	-	-	-	1	0.5
Itching	5	2.5	4	2.0	1	0.5	-	-	10	5.0
Eye pain	8	4.0	5	2.5	-	-	1	0.5	14	7.0
Tearing	10	5.0	1	0.5	1	0.5	-	-	12	6.0
Blurred vision	11	5.5	2	1.0	1	0.5	1	0.5	15	7.5
Light sensitivity	11	5.5	4	2.0	1	0.5	1	0.5	17	8.5
Eye congestion	2	1.0	-	-	-	-	-	-	2	1.0
Eye secretions	4	2.0	-	-	-	-	-	-	4	2.0

No significant correlation was noted between experiencing COVID-19-related ocular symptoms and previous medical or ocular history. A weak positive significant correlation was noted between experiencing COVID-19-related ocular symptoms and the number of times an individual was infected with COVID-19 (Table 4). In terms of age groups and gender, no significant correlation was noted.

**Table 4:** Spearman's correlation analysis (N=200)

Variable	Statistic	1	2	3
	r_s_	1		
1. Experienced ocular symptom (Yes, No)				
	p-value			
	r_s_	-0.005	1	
2. Prior diagnosis with a medical condition (Yes, No)				
	p-value	0.984		
3. Number of times infected with COVID-19 (once, twice, thrice, more)	r_s_	0.219^**^	0.099	1
	p-value	0.002	0.162	

## Discussion

Our study shows that 20.5% of COVID-19 patients experience ocular symptoms. Other studies have presented different prevalence and incidence rates of ocular symptoms. It is possible that during the disease course of COVID-19, ophthalmic manifestations can develop at any point, and the patients may not be able to recall those symptoms [1-3]. The ocular surface is considered to be highly vulnerable to the entry of the COVID-19 virus in addition to the conventional respiratory route. The entry of the virus through the ocular surface could reflect the diversity in ocular manifestations reported in patients with COVID-19. Previous studies have shown that 2%-64% of COVID-19 patients experience ocular manifestations [12].

Among patients who experienced ocular symptoms in our study, light sensitivity (8.5%) and blurred vision (7.5%) were the most common. The study by McHarg M et al. also reported light sensitivity, eye redness, and blurred vision as the most common ocular symptoms in patients, with the percentages reaching up to 31.0% [13]. Recent meta-analyses by Soltani et al. and Nasiri et al. found that ocular surface symptoms are predominant when eye symptoms are present, suggesting a milder ocular disease course than when COVID-19 affects other systems [12,14]. However, some researchers have reported serious ocular complications that could lead to blindness as well [11,15-17].

Our study suggested that patients with recurrent COVID-19 infection may be at a greater risk of experiencing COVID-19-related ocular symptoms; however, a weak significant association was noted between the frequency of COVID-19 and the occurrence of ocular symptoms. Other variables such as gender, age, and medical and ocular history did not significantly correlate with the development of ocular symptoms. Only 12% of patients who experienced ocular symptoms sought medical care by an ophthalmologist. This finding is comparable to that reported in a study by McHarg M. et al. [13]. The low percentage of patients seeking medical attention by an ophthalmologist suggests that ocular symptoms related to COVID-19 are often mild and are usually resolved spontaneously.

The study has several limitations. The number of participants was relatively small. Moreover, the survey was not administered to patients who had an acute presentation of the disease course, and this could influence the recalling ability of the participants. Such a recall bias is typically unavoidable in a survey study. Furthermore, the association between systemic and ocular manifestations was not investigated in line with the severity of the disease. Further research is required to understand the pathophysiology and the course of ocular symptoms associated with COVID-19.

## Conclusions

We found that about one-fifth of patients with COVID-19 in the Qassim region of Saudi Arabia experienced ocular symptoms, which were mostly mild, and few sought medical attention. These findings suggest that ocular symptoms related to COVID-19 are often underreported and may not require medical intervention in many cases. However, given the potential for viral entry through the ocular surface, it remains important to monitor such symptoms, especially in recurrent cases. Further studies should investigate the role of ACE2 receptors in ocular tissues and the association of recurrent infections with ocular symptoms. More research is also necessary to explore whether the severity of systemic COVID-19 correlates with more severe ocular manifestations. This would help in understanding the full scope of COVID-19-related ocular issues and inform better clinical practices.

## Appendices

Appendix 1

Data Collection Sheet

Demographic data

Name:

Age:

Gender:

Medical history

**Table 5:** Medical history

Diabetes:	Yes	No
Hypertension:	Yes	No
Smoker:	Yes	No
Infected more than once:	Yes	No
Admitted in hospital:	Yes	No
Eye procedure:	Yes	No

Covid-19 related eye symptoms

Have you had any eye symptoms?

A) Yes

B) No

**Table 6:** Ophthalmological symptoms

If yes, what are the symptoms?		
Dryness?	Yes	No
Redness?	Yes	No
Eye discharge?	Yes	No
Foreign body sensation?	Yes	No
Burning sensation?	Yes	No
Conjunctival congestion?	Yes	No
Double vision?	Yes	No
Itching?	Yes	No
Eye pain?	Yes	No
Photophobia?	Yes	No
Tearing?	Yes	No
Blurred vision?	Yes	No
Others?	……………………………	

Did you seek medical attention by eye doctor?

A) Yes

B) No
